# Adult conjunctivitis secondary to dual infection with *Chlamydia trachomatis* and *Neisseria gonorrhoeae* - A case report

**DOI:** 10.1016/j.ajoc.2018.11.009

**Published:** 2018-11-14

**Authors:** Emma Linton, Lisa Hardman, Lynn Welburn, Imran Rahman, Jaya Devi Chidambaram

**Affiliations:** a Dept. of Ophthalmology, Blackpool Victoria Hospital, Whinney Heys Road, Blackpool, FY3 8NR, UK; b Manchester Royal Eye Hospital, Oxford Road, Manchester, M13 9WL, UK; c Dept. of Microbiology, Blackpool Victoria Hospital, Whinney Heys Road, Blackpool, FY3 8NR, UK; d Dept. of Clinical Research, London School of Hygiene and Tropical Medicine, Keppel Street, London, WC1E 7HT, UK

**Keywords:** *Chlamydia trachomatis*, *Neisseria gonorrhoeae*, Conjunctivitis, Adult inclusion conjunctivitis, Sexually transmitted disease, Bacterial conjunctivitis

## Abstract

**Purpose:**

Although *Chlamydia trachomatis* and *Neisseria gonorrhoeae* are the commonest sexually transmitted infections in England, reports of ocular co-infection in the literature are limited. We report such a case which responded well to treatment, and discuss the literature and evidence currently available with regards to management of these cases.

**Observations:**

The patient is a 48-year-old bisexual gentleman who presented to the eye clinic of a UK hospital with redness, discharge and blurred vision in his left eye for one week. Initially he had mucopurulent discharge but his cornea was clear. He did not comply with prescribed treatment and returned two days later with bilateral symptoms and corneal thinning in his left eye peripherally.

PCR tests for *Chlamydia trachomatis* and *Neisseria gonorrhoeae* were positive and the patient was commenced on intravenous ceftriaxone, oral and topical levofloxacin eye drops. After 48 hours of inpatient treatment the patient showed clinical improvement.

**Conclusions and importance:**

Ophthalmologists should be aware of the possibility that *Chlamydia trachomatis* and *Neisseria gonorrhoeae* can cause co-infection in adult conjunctivitis, and of the straightforward method of treatment for such individuals. Delayed diagnosis and treatment of affected patients can lead to corneal complications and potential blindness. It is advisable to discuss these cases with the local microbiology service wherever possible, and referral to a sexual health service is imperative.

## 1 Introduction

*Chlamydia trachomatis* and *Neisseria gonorrhoeae* are the two most common sexually transmitted infections in England.^1^ Both can cause conjunctivitis in adults that can be easily treated if recognized early. Reports of ocular co-infection causing conjunctivitis in adults are scarcely reported in the literature,^2^ but can have serious sight-threatening consequences if diagnosis is not made promptly and correct treatment initiated. We report a case of conjunctivitis due to dual infection with *Chlamydia trachomatis* and *Neisseria gonorrhoeae*, which responded well to treatment.

## 2 Case report

A 48-year-old bisexual gentleman presented to the eye clinic with a one week history of redness, discharge and reduced vision in his left eye. He had no past ocular history, and no recent systemic upset. He had a background of schizophrenia and obsessive compulsive disorder for which he received zuclopenthixol intramuscular injections every three weeks. Recent sexual history revealed multiple sexual partners, both male and female, without use of protection.

On examination visual acuity unaided was 0.20 LogMAR right eye (improving with pinhole suggesting untreated refractive error) and Hand Movements (HM) in the left eye, no improvement with pinhole. Anterior segment examination revealed profuse mucopurulent discharge from the left eye, with upper and lower eyelid swelling and conjunctival injection. The cornea was clear. The right anterior segment was normal. Sterile swabs were used to obtain conjunctival surface samples from the left eye for bacterial culture (Sterilin Charcoal Transport Swab, Thermo Fisher Scientific, Loughborough, UK), for Herpes Simplex Virus PCR testing (Remel, Lenexa, USA) and *Chlamydia trachomatis* testing (COBAS PCR Dual Media Swab, Roche Diagnostics Limited, West Sussex, UK). The patient was commenced on 2 hourly Ofloxacin 0.3% eye drops and chloramphenicol 1% ointment 4 times daily and discharged home.

**Fig. 1:**
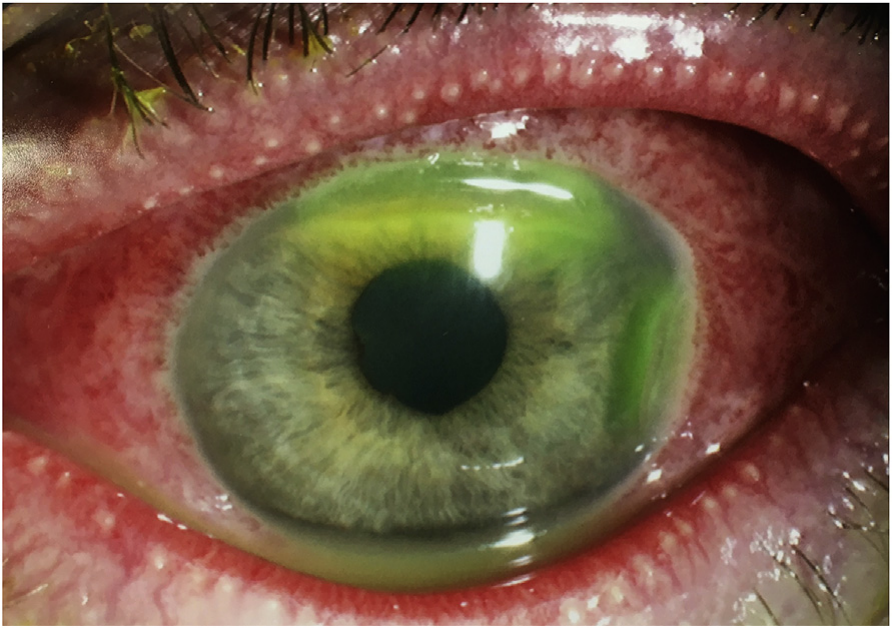
Anterior segment slit lamp photograph of the patient's left eye, showing two regions of corneal thinning adjacent to the limbus, at superior and temporal cornea. The mucopurulent discharge is also evident in the tear film.

**Fig. 2:**
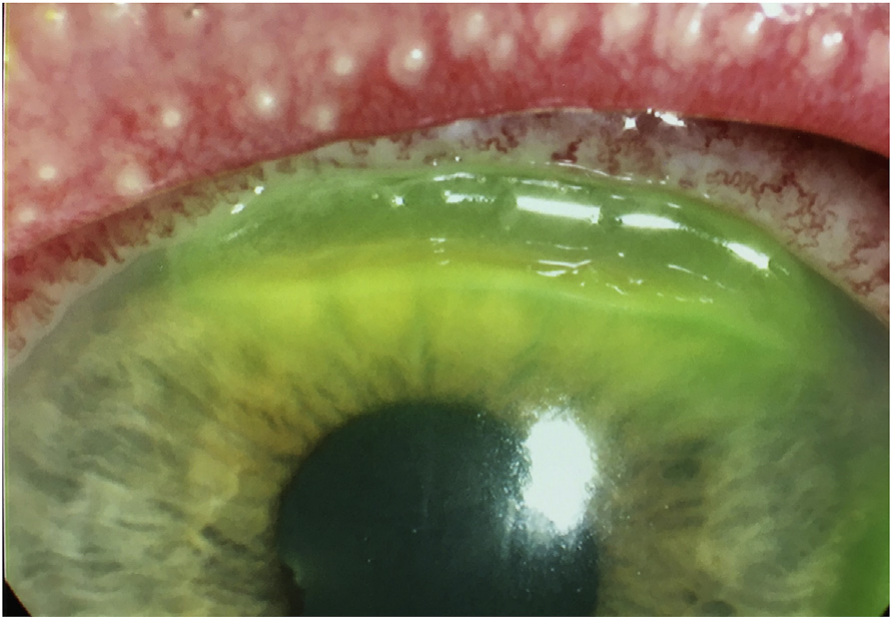
Anterior segment slit lamp photograph of the patient's left eye focusing on the area of superior thinning of the cornea.

The patient returned two days later with worsening symptoms that were now present bilaterally. He had not used any of the prescribed eyedrops. Examination of the anterior segment showed bilateral profuse mucopurulent discharge, lid swelling and conjunctival injection. The right cornea was clear but the left cornea now showed two areas of thinning peripherally in the superior and temporal regions (Fig. 1 and Fig. 2). Due to compliance issues with initial treatment and worsening of the clinical signs, the patient was admitted to the ward for further investigations and management.

**Fig. 3:**
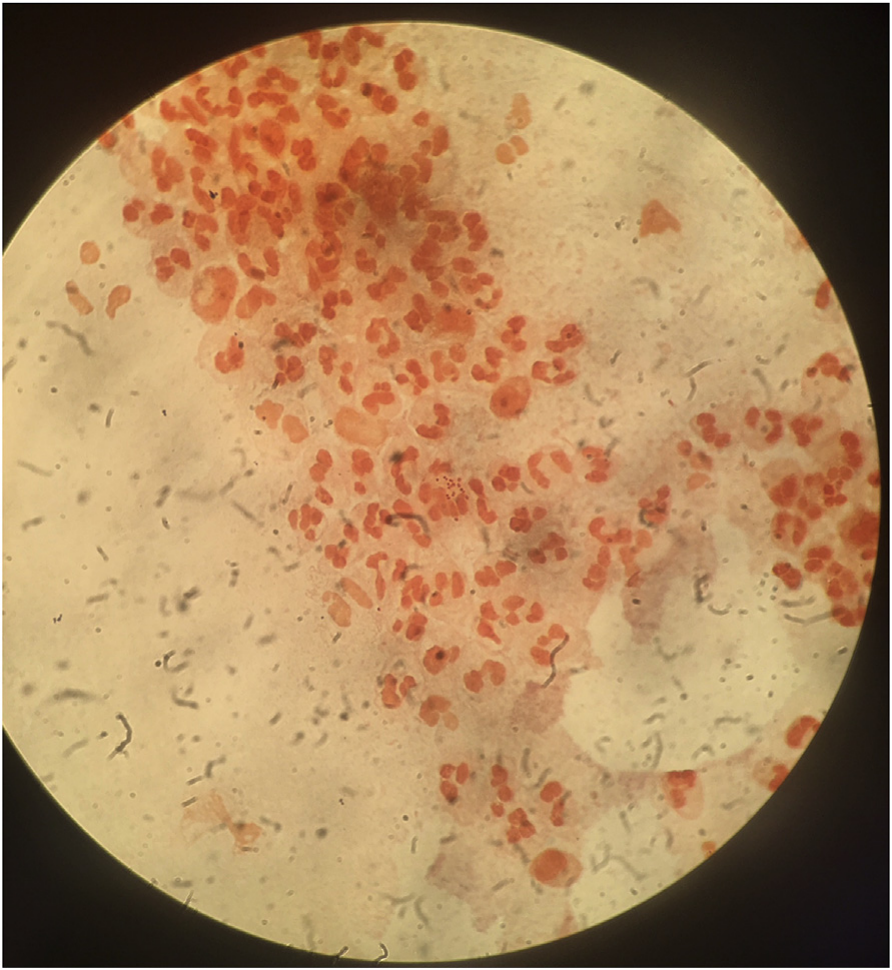
Gram film from the patients conjunctival surface sample, showing central cluster of gram negative diplococci with surrounding polymorphonuclear leukocytes.

Initial test results were positive for *Chlamydia trachomatis* and the clinical suspicion of co-infection with *Neisseria gonorrhoeae* led to further samples being taken. These were conjunctival surface swab samples from the everted upper lid and lower lid, that were directly placed on to sterile glass slides for Gram stain, followed by inoculation of chocolate agar and blood agar plates. An additional conjunctival swab sample (Transwab, Medical Wire, Wiltshire, UK) was sent for polymerase chain reaction (PCR) for *Neiserria gonorrhoeae* testing (COBAS PCR Dual Media Swab Kit, Roche Diagnostics Limited, West Sussex, UK). The gram stain was performed according to standard procedures and showed gram-negative diplococci consistent with a diagnosis of *N. gonorrhoeae* infection (Fig. 3), later confirmed by a positive PCR test result.

Following a discussion with the microbiologist the patient was commenced on intravenous ceftriaxone 2mg once daily, oral azithromycin 500mg once daily for five days and topical levofloxacin preservative-free drops to both eyes hourly.

48 hours after commencing treatment there were signs of clinical improvement. Visual acuity had risen to logMAR 0.50 in the left eye, unaided. Corneal thinning had stabilized and re-epithelialization of the thinned cornea was observed. The patient was referred to the Genito-urinary Medicine team for systemic test of cure and contact tracing.

## 3 Discussion

*Neisseria gonorrhoeae* are gram-negative diplococci which can infect virtually any mucous membrane in the body. It is the second most common bacterial sexually transmitted infection (STI) in England and was diagnosed three times more commonly in men than women in 2016.^1^^,^^3^ Gonococcal conjunctivitis is rare and specific nationwide data on the incidence is not reported at present. It is usually transmitted by direct spread from the genitalia and has an acute onset of symptoms. Signs include lid swelling, a profuse purulent discharge, keratitis and pre-auricular lymphadenopathy. Although rare, corneal involvement can occur and lead to globe perforation.^4^^,^^5^

*Chlamydia trachomatis* is a gram-negative bacteria and the commonest bacterial STI worldwide.^6^ Subtypes of *Chlamydia trachomatis* are associated with different presentations. Serovars A-C cause trachoma which is a disease endemic in several developing countries that causes conjunctival scarring, trichiasis, corneal ulceration and blindness.^7^ Subtypes D-K of *Chlamydia trachomatis* cause the STI and adult inclusion conjunctivitis.^7^ It is estimated that *Chlamydia trachomatis* is the organism responsible for 20% of cases of adult bacterial conjunctivitis.^8^ It usually infects the eye via direct spread from infected genitalia and has an incubation period of around one week. Signs include discharge, follicular conjunctivitis, superior corneal pannus and a pre-auricular lymph node.^8^

To the best of our knowledge there has only been one case of conjunctivitis caused by co-infection with *N. gonorrhoeae* and *Chlamydia* sp. in an adult documented in the literature.^2^ However, corneal involvement has been estimated to occur in around 34% of patients with gonococcal conjunctivitis and can rapidly lead to perforation if not treated promptly and correctly.^9^ Risk factors for perforation include older age and delayed presentation.^9^ For this reason, it is important that ophthalmologists who see patients with a severe conjunctivitis that does not respond to first-line broad-spectrum topical antibiotics (e.g. chloramphenicol eyedrops) and with signs of corneal involvement should have a high index of suspicion for gonococcal infection and investigate patients accordingly even if they have already tested positive for *Chlamydia* sp. The differential diagnosis for an adult patient presenting with mucopurulent discharge should include conjunctivitis secondary to bacteria (such as *Staphylococcal* sp., *Streptococcal* sp., *Haemophilus influenza*, *Moraxella* sp., *Chlamydia* sp., and *Neisseria* sp.), viruses (e.g. adenovirus) as well as allergic or and toxic conjunctivitis.

There are currently no specific guidelines for the treatment of conjunctival co-infection with *Chlamydia* sp. and *N. gonorrhoeae* in adults in the UK. However, systemic treatment for non-ocular co-infection is recommended by the British Association for Sexual Health and HIV (BASHH) using ceftriaxone 500mg intramuscularly and 1g azithromycin orally. Similar approaches for both chlamydial and gonococcal conjunctivitis are reported in the literature with the vast majority of cases responding well to these agents.^10^, ^11^, ^12^, ^13^ In the United States the American Academy of Ophthalmology (AAO) published a Preferred Practice Pattern for Conjunctivitis.^14^ They recommend that adults treated for gonococcal conjunctivitis be routinely treated with medication effective against Chlamydia trachomatis because patients are often co-infected. They suggest Azithromycin 1g orally as a single dose or Doxycycline 100mg orally, twice a day for 7 days. This guidance is mirrored by the US Centre for Disease Control and Prevention (CDC).^15^

On 7th July 2017 the World Health Organisation published a press release stating concerns about the increasing worldwide resistance of *N. gonorrhoeae* to anti-microbial drugs.^16^ Their global data, collected from 2009 to 2014, showed 97% of countries had identified *N. gonorrhoeae* strains resistant to ciprofloxacin, 81% had detected resistance to azithromycin and 66% had found resistance for extended-spectrum cephalosporins such as ceftriaxone.^13^ This is of great concern as there is little research focusing on the development of new anti-microbial drugs to combat *N. gonorrhoeae*.^16^ Ophthalmologists can help to limit this growing resistance by treating patients only if they are microbiologically-positive for *N. gonorrhoeae* and counselling patients about the importance of completing the course of antibiotics prescribed, in order to reduce the development of bacterial resistance.

## 4 Conclusion

Ophthalmologists should be aware of the possibility that *Chlamydia trachomatis* and *Neisseria gonorrhoeae* can cause co-infection in adult conjunctivitis, and of the straightforward method of treatment for such individuals. Delayed diagnosis and treatment of affected patients can lead to corneal complications and potential blindness. It is advisable to discuss these cases with the local microbiology service wherever possible, and referral to a sexual health service is imperative.

## Patient Consent

Consent to publish this case report could not be obtained as the patient was lost to follow-up. This report does not contain any personal information that could lead to the identification of the patient.

## Funding

Wellcome Trust grant no. 097437/Z/11/Z to JDC. The funder had no role in the study design, data analysis or result interpretation.

## Conflicts of interest

The authors have no financial disclosures.

## Authorship

All authors attest that they meet the current ICMJE criteria for Authorship.

## Appendix A Supplementary data

Data Profile Data Profile
